# Pyrite-Based Cr(VI) Reduction Driven by Chemoautotrophic Acidophilic Bacteria

**DOI:** 10.3389/fmicb.2019.03082

**Published:** 2020-02-07

**Authors:** Xinxing Liu, Haiyan Wu, Min Gan, Guanzhou Qiu

**Affiliations:** Department of Biology, Key Laboratory of Biohydrometallurgy of Ministry of Education, School of Minerals Processing and Bioengineering, Central South University, Changsha, China

**Keywords:** pyrite, Cr(VI) reduction, *Acidithiobacillus ferrooxidans*, pH, passivation effect

## Abstract

Cr(VI) is considered as a priority pollutant, and its remediation has attracted increasing attention in the environmental area. In this study, the driving of pyrite-based Cr(VI) reduction by *Acidithiobacillus ferrooxidans* was systematically investigated. The results showed that pyrite-based Cr(VI) reduction was a highly proton-dependent process and that pH influenced the biological activity. The passivation effect became more significant with an increase in pH, and there was a decrease in Cr(VI) reduction efficiency. However, Cr(VI) reduction efficiency was enhanced by inoculation with *A. ferrooxidans*. The highest reduction efficiency was achieved in the biological system with a pH range of 1–1.5. Pyrite dissolution and reactive site regeneration were promoted by *A. ferrooxidans*, which resulted in the enhanced effect in Cr(VI) reduction. The low linear relevancy between pH and Cr(VI) dosage in the biological system indicated a complex interaction between bacteria and pyrite. Secondary iron mineral formation in an unfavorable pH environment inhibited pyrite dissolution, but the passivation effect was relieved under the activity of *A. ferrooxidans* due to S/Fe oxidization. The balance between Cr(VI) reduction and biological activity was critical for sustainable Cr(VI) reduction. Pyrite-based Cr(VI) remediation driven by chemoautotrophic acidophilic bacteria is shown to be an economical and efficient method of Cr(VI) reduction.

## Introduction

Hexavalent chromium [Cr(VI)] is an important raw material that is widely applied in various industries including in metallurgy, electroplating, and leather tanning. Two forms of Cr with different oxidation states, Cr(VI) and Cr(III), usually exist in the natural environment. Cr(VI) is also considered a priority pollutant, and its remediation has attracted increasing attention in the environmental area (Jiang et al., 2015). The toxicity of Cr(III) is far lower than that of Cr(VI), and it can be easily precipitated as Cr(OH)_3_, while Cr(VI) is soluble over a wide pH range. Reduction represents an efficient strategy for Cr(VI) remediation (Parker et al., 2011; Huggins et al., 2016).

The potential of sulfide minerals such as pyrite or reduced sulfur in redox-sensitive contaminant remediation has attracted great interest in recent years (He and Traina, 2005). However, the pyrite surface is usually passivated due to the formation of Fe-Cr compounds, resulting pyrite utilization being blocked and thus inhibiting further Cr(VI) reduction (He and Traina, 2005; Gong et al., 2017). Strengthening measures that have been applied in pyrite or iron-containing material-based Cr(VI) reduction mainly focus on thermal modification, mechanical crushing, and organic acid/chelating agent addition, etc. (Doyle et al., 2004). Physical treatment increases the specific surface area and degree of pyrite reaction, which enhances Cr(VI) reduction efficiency. Organic acid and chelating agents act as buffers in the reaction, contributing to the maintenance of a suitable pH range (Kantar et al., 2015; Kantar and Bulbul, 2016; Mandal et al., 2017). The pyrite surface is property modified after these treatments, and the passivation is also relieved to some extent. However, Cr(VI) reduction still remains at an unsatisfactory level, as only the pyrite surface is involved in the reduction reaction. Fast and orderly mineral dissolution determines whether pyrite-based Cr(VI) reduction can be utilized at an industrial scale. According to our previous research, the activity of *Acidithiobacillus ferrooxidans* significantly enhanced sulfur oxidization and pyrite dissolution as well as the corresponding Cr(VI) reduction (Gan et al., 2018; Wang et al., 2019).

It should be noted that the influence of pH on pyrite-based Cr(VI) reduction remains unclear, especially in the complex system where *A. ferrooxidans* is present. The chemical equilibrium and the oxidative properties of Cr, Fe, and S in solution are mainly determined by protons (Kim et al., 2002; Bae and Hanna, 2015). Protonation/deprotonation, pyrite dissolution, and Cr(VI) adsorption in the Cr(VI) reduction reaction are influenced by pH. The surface properties of the pyrite will change with pH variation. In addition, biological activities, including Fe/S metabolism, bacteria proliferation, and physiological activity, are also affected by pH. A higher Cr(VI) reduction efficiency can be obtained in the optimal pH range in practical applications. The purposes of this study are to analyze the synergistic effect between acidophilic bacteria and pyrite in Cr(VI) reduction, to shed light on the effect of pH and dosage on pyrite-bacteria-Cr(VI) interaction, and to contribute to an increase in pyrite dissolution and Cr(VI) reduction efficiency. This research is of environmental and practical significance to solve redox-sensitive pollution problems in the mining and smelting industries.

## Materials and Methods

### Microorganism, Medium, and Culture Conditions

The acidophilic bacterium *A. ferrooxidans* 23,270 used in this study was kept at the Key Laboratory of Biometallurgy of Ministry of Education, China. It was cultured in 9 K medium with the addition of 10 g/L S^0^ as an energy source at 30°C and at 180 rpm. Sublimed sulfur with a purity higher than 99.5% was purchased from TianJing Hengxing Chemical Reagent Co. Ltd. The composition of the 9 K medium was as follows: (NH_4_)_2_SO_4_ 3.0 g/L, KC1 0.1 g/L, K_2_HPO_4_ 0.5 g/L, MgSO_4_ 0.5 g/L, and Ca(NO_3_)_2_ 0.01 g/L. The medium was adjusted to pH 2.0 with dilute sulfuric acid and autoclaved for 20 min at 121°C. All reagents were of analytical grade. Bacteria were harvested at the end of the exponential phase (about 4 days). Cultures were first filtered by 0.45 μm filter paper to remove the precipitate. The filtrate was then centrifuged at 12,000 rpm for 20 min to harvest the cells. Cells were washed twice and resuspended in distilled water.

### Cr(VI) Reduction Experiment

The experiment on the influence of pH on Cr(VI) reduction was conducted in a 500-ml conical flask with 75 ml 9 K medium and 2 g pyrite. Natural pyrite was purchased from the geological museum of Guangxi Province, China; this contained 47.3% Fe and 48.4% S (wt%). In the pH-influence experiment, Cr(VI) was added daily (potassium chromate, 1,000 mg/L) depending on the reductive substance in the reaction solution, which was determined by diphenylcarbazide (DPCI) chromogenic reaction. If the reductive substance was fully consumed, no further Cr(VI) solution was added. Diphenylcarbazide chromogenic reaction was used to judge whether Cr(VI) was completely reduced to Cr(III). The diphenylcarbazide reaction system would turn to purple if the reductive substance was fully consumed by Cr(VI). The experiment was divided into four groups, namely the pH-stable biological group (BS), the pH-stable chemical group (CS), the pH-independent biological group (BI), and the pH-independent chemical group (CI). The pH of independent systems was only adjusted in the initial stage, while the pH of stable systems was adjusted with 5% H_2_SO_4_ (v/v) every 2 days. In the pH-independent systems, pH was set at 0.5, 1.0, 1.5, 2.0, 2.5, 3.0, and 3.5. In the pH-stable systems, pH was set in the ranges 1–1.5, 1.5–2, 2–2.5, 2.5–3, and 3–3.5. *A. ferrooxidans* was inoculated into the system with initial cell density at 1 × 10^8^/ml, which was determined by the microscopic count method. Cr(VI) solution was added from the second day in both biological and chemical treatments in order to enhance the adaptability of *A. ferrooxidans*.

In the experiment on the influence of Cr(VI) dosage, Cr(VI) solution was added to the system in gradients of 0.5, 2, 4, 6, and 8 mg per day (potassium chromate, 1,000 mg/L). In the dosage-dependent experiment (D), Cr(VI) was continually added to the conical flask until all of the reductive substance was consumed every day, using the same addition method as in the pH influence experiment. The reaction was transferred to a bigger conical flask if the reaction volume exceeded 2/3 of the container volume. All of the experiments above were carried out in duplicate.

### Physicochemical Parameters of the Reduction Determination

A pH meter (PHS-3C) with a Pt electrode using a calomel electrode as a reference was employed to measure the pH and redox potential of the reduction system. The concentration of Fe(II) and total iron in solutions was determined by using a modified 1, 10-phenanthroline method (Harvey et al., 1955). Cr(VI) concentration was detected by the 1,5-diphenyl-carbazide method after centrifuging the supernatant for 5 min at 12,000 rpm. Morphological analysis was conducted using an FEI Electron Optics field emission scanning electron microscope (Nova NanoSEM230) operated at 15 kV accelerating voltage. Samples were coated with a thin layer of gold in a Pelco Model 3 Sputter 91,000 coater. The precipitate used for XPS analysis were taken at the end of the reaction. First, they were quickly washed with pH 2.0 9 K medium, and they then underwent freeze vacuum drying for 24 h. X-ray photoelectron spectroscopy analysis was conducted with a Thermo Fisher X-ray photoelectron spectrometer (model ESCALAB 250Xi). Spectra were recorded at a constant pass energy of 20 eV and 0.1 eV/step using an Al Ka X-ray source. Binding energies were relative to the C 1 s level at 284.8 eV. The spectra of S(2p) and Fe(2p) were used to investigate the oxidation states and species. The spin-orbit doublets S2p_3/2_–S2p_1/2_ showed a splitting energy of 1.19 eV. The peaks were resolved using CasaXPS version 2.3.18 assuming that the two components had equal peak widths and relative peak areas of 2:1.

## Results and Discussion

### Pyrite Dissolution in the Proton Compensation System

**Figure 1:**
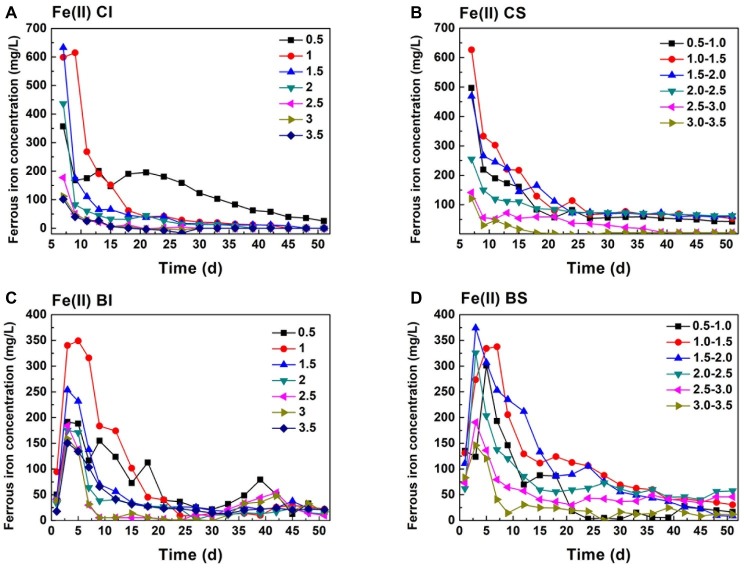
Variation of ferrous iron in pH-stable and pH-independent system; (**A**, CI): pH-independent chemical group, (**B**, CS): pH-stable chemical group, (**C**, BI): pH-independent biological group, (**D**, BS): pH-stable biological group.

According to the stoichiometry, Cr(VI) reduction is a proton consumption reaction. A total of 7 mol H^+^ is consumed in 1 mol Cr(VI) reduction when Fe(II) serves as the reductant (Eq. 1), and 4.8 mol H^+^ is required in FeS_2_-based Cr(VI) reduction (Eq. 2; Kim et al., 2001; Lin and Huang, 2008). It can be speculated that Cr(VI) reduction performance is highly correlated with pH variation. Pyrite dissolution was mainly caused by two factors, Fe(III)/Cr(VI)-dominated chemical oxidation and *A. ferrooxidans*-mediated biological oxidation (Demoisson et al., 2005, 2007; Lara et al., 2015). The Fe species was an indicator for pyrite dissolution and also the key reductant for Cr(VI) reduction. The Fe(II) concentration initially exceeded 600 mg/L in pH 1.0 and 1.5 in the CI system, while it decreased to ca. 350 mg/L initially at an pH of 0.5 (Figure 1). With continuous Cr(VI) dosing, the Fe(II) concentration decreased drastically and approached equilibrium after 10 days, indicating that pyrite dissolution and Fe(II) regeneration were inhibited under high pH. In the chemical system, proton dependent Fe(III) or Cr(VI) chemical attack led to pyrite dissolution. It can be identified from Figure 1 that the Fe(II) concentration in the BI system was lower than that in the CI system, which was caused by the biological oxidization of Fe(II) by *A. ferrooxidans* (Zhu et al., 2013; Christel et al., 2018). This would have adverse effects on Fe(II)-based Cr(VI) reduction, but it might promote pyrite dissolution. In the BS system, the Fe(II) concentration remained at 80 mg/L in the middle and later periods, significantly higher than that in the CI system, which indicated that proton compensation promoted pyrite dissolution. According to previous research, Cr(VI) reduced to Cr(III) in two ways: intermediate sulfur and Fe(II) released from pyrite dissolution and active sites on pyrite (QuiIntana et al., 2001; Bae and Hanna, 2015; Li et al., 2016). Therefore, both reduction pathways should be taken into account in practical applications to enhance the reduction efficiency.

(1) $$6Fe_{\left(aq\right)}^{2+}+Cr_{2}O_{7\;\left(aq\right)}^{2-}+14H_{\left(aq\right)}^{+}\to 6Fe_{\left(aq\right)}^{3+}+2Cr_{\left(aq\right)}^{3+}+7H_{2}O$$

(2) $$3FeS_{2}+15HCrO_{4}^{-}+57H^{+}\overset{A.f}{\to }3Fe^{3+}+6SO_{4}^{2-}+15Cr^{3+}+36H_{2}O$$

(3) $$E=E^{0}+\frac{\mathrm{nF}}{RT}\ln\left(Fe^{3+}/Fe^{2+}\right)$$

(4) $$FeS_{2}+8H_{2}O+14Fe^{3+}\to 15Fe^{2+}+2SO_{4}^{2-}+16H^{+}$$

**Figure 2:**
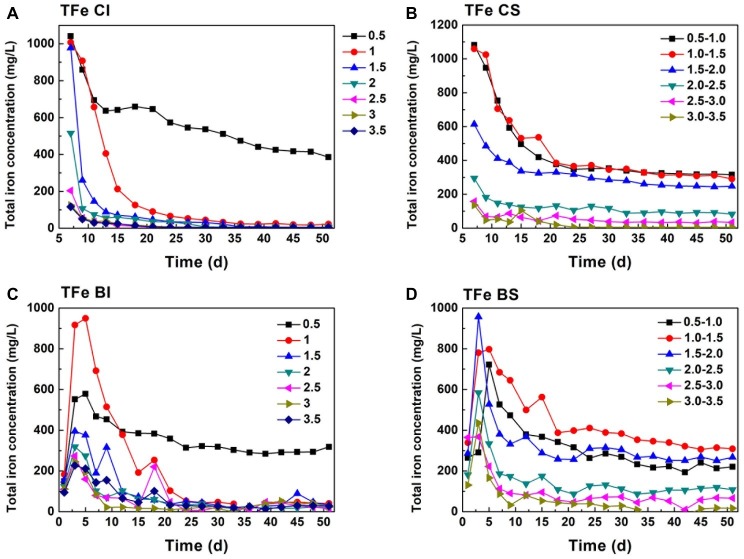
Total iron change in pH-stable and pH-independent system; (**A**, CI): pH-independent chemical group, (**B**, CS): pH-stable chemical group, (**C**, BI): pH-independent biological group, (**D**, BS): pH-stable biological group.

Comparing Fe(II) (Figure 1) and TFe (Figure 2) in the systems, it can be found that Fe mainly existed in the form of Fe(II). TFe in the CI pH 0.5 system was higher than 400 mg/L during the whole reduction. The high Fe(III) concentration contributed to a high redox potential (Eq. 3) and promoted pyrite oxidation dissolution (Eq. 4). Fe decreased rapidly with an increase in pH and may have been precipitated in secondary iron minerals such as jarosite (Zhu et al., 2013; Gan et al., 2015). The deposition of secondary iron minerals on the pyrite surface would have been unfavorable for pyrite dissolution (Stott et al., 2000; He and Traina, 2005). Basically, the TFe concentration in the biological system was lower than that in both the pH-independent and -stable chemical treatments. This indicated that more passivation substances formed under the activity of *A. ferrooxidans*. TFe in the pH-stable system decreased in the initial stage and entered into a stable phase after 20 days and was significantly higher than that in pH-independent group. A stable and low pH promoted pyrite decomposition.

**Figure 3:**
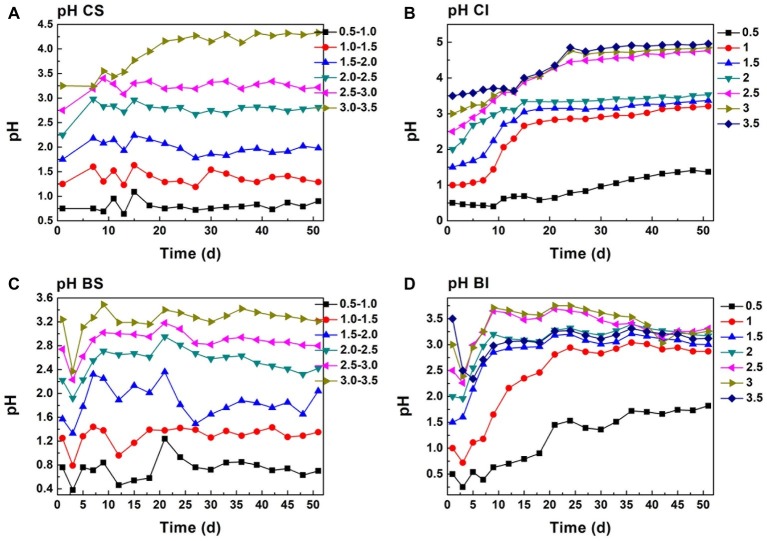
pH change in proton compensation and un-compensation system; (**A**, CS): pH-stable chemical group, (**B**, CI): pH-independent chemical group, (**C**, BS): pH-stable biological group, (**D**, BI): pH-independent biological group.

As analyzed above, Cr(VI) reduction was a proton-consumption reaction, while protons can be regenerated in the pyrite biological oxidization process. Moreover, the pH of the system is maintained in a specific interval under exogenetic proton compensation. Hence, pH variation was a comprehensive reflection of Cr(VI) reduction, biological oxdization, and proton compensation. It showed that the pH of proton compensation systems was kept in a stable interval (Figures 3A,C). The pH of the CS system was maintained basically at 0.8, 1.4, 2.0, 2.9, 3.4, and 4.4, and pH of the corresponding biological system was 0.8, 1.2, 1.6, 2.5, 3.0, and 3.3 because of the protons generated in sulfur oxidation by *A. ferrooxidans*. This phenomenon also manifested the dissolution effect of *A. ferrooxidans* on pyrite (Eq. 5). When *A. ferrooxidans* induced 1 mol FeS_2_ dissolution, 1 mol Fe(II) and 2 mol H^+^ would be produced, resulting in the regeneration of proton and reactive sites as well as sustainable Cr(VI) reduction (Tu et al., 2017). An obvious rise in pH could be observed in the CI and BI systems (Figures 3B,D). It reached 1.0, 2.8, 2.9, 3.1, 4.5, 4.5, and 4.6 at the endpoint in CI systems, but the corresponding pH in BI systems was 1.5, 2.7, 2.8, 3.0, 3.0, 3.1, and 3.1 respectively. This indicated the effective pyrite dissolution and proton regeneration under the activity of *A. ferrooxidans*, which was critical for Cr(VI) reduction.

(5) $$2FeS_{2}+2H_{2}O+7O_{2}\overset{A.f}{\to }2Fe^{2+}+4SO_{4}^{2-}+4H^{+}$$

The redox potential was a comprehensive reflection of a series biogeochemical processes including pyrite dissolution, Fe(II) oxidization, and secondary iron mineral formation (Sun et al., 2015). The redox potential was maintained at around 420 mV in the initial pH 0.5 CI system, and it decreased to 300 mV in the initial pH 3.5 system (Supplementary Figure S1). Redox potential decreased as pH increased with no proton compensation, and this occurred in both biological and chemical systems. The protons produced by acidophilic bacteria in pyrite dissolution and sulfur oxidization cannot replenish the protons consumed by Cr(VI) reduction. Redox potential in pH-stable treatment was obviously higher than that with pH-independent treatment. Redox potential in the CS pH 0.5–1.0 system reached 480 mV, and it decreased to 320 mV with an increase in pH. Fe(III) and protons will have attacked pyrite directly and promoted pyrite dissolution.

### Cr(VI) Reduction Behavior

**Figure 4:**
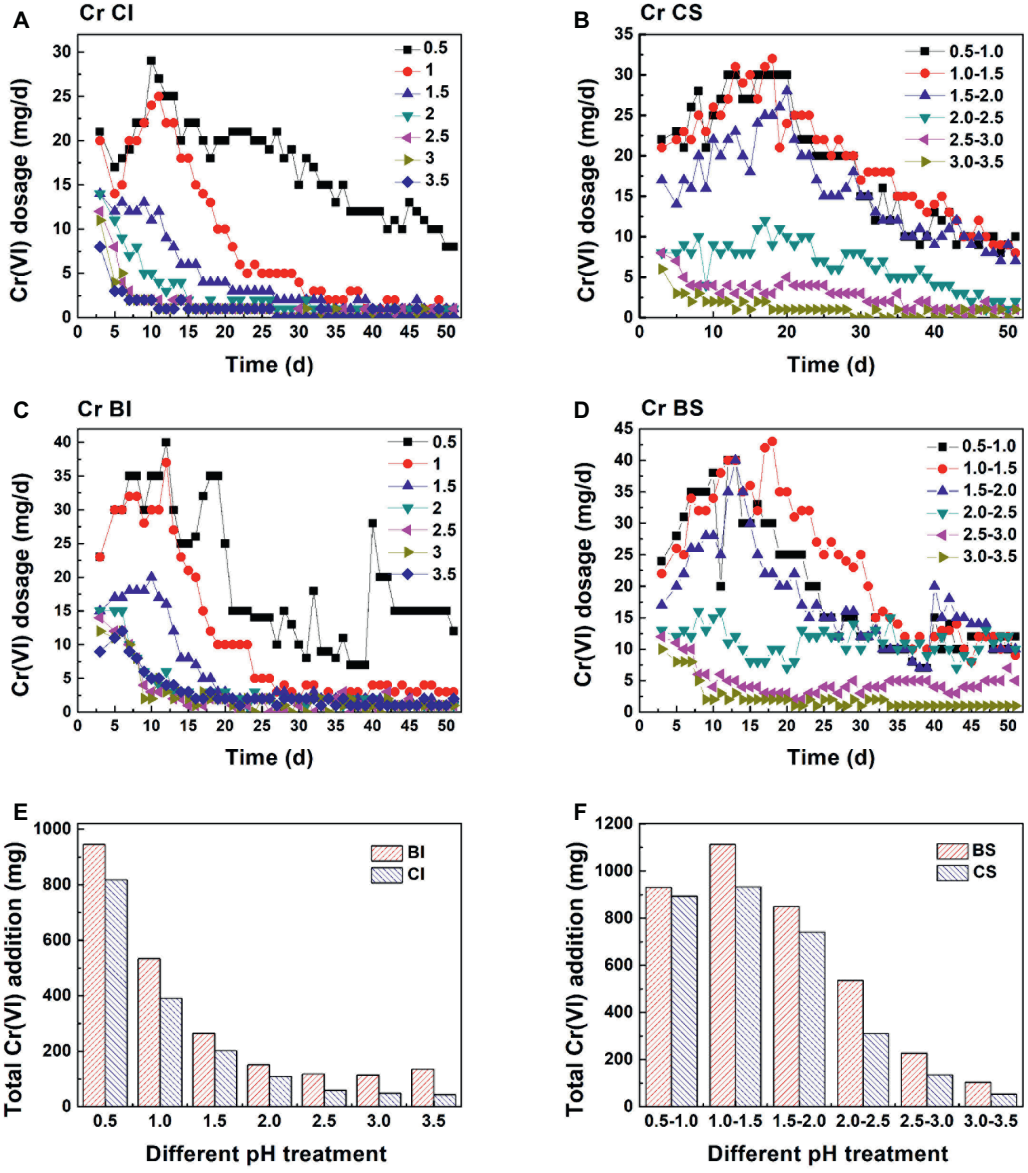
Cr(VI) reduction behavior in different systems: Cr(VI) daily reduction behavior in the pH-independent chemical group (**A**, CI), pH-stable chemical group (**B**, CS), pH-independent biological group (**C**, BI), and pH-stable biological group (**D**, BS); total amount of Cr(VI) reduction in the pH-independent **(E)** and -stable **(F)** systems.

Cr(VI) reduction in the CI system was basically negatively correlated with pH (Figure 4). The Cr(VI) reduction dosage increased steadily in pH 0.5 and 1.0 (CI and BI system) during the first 12 days. The reduction dosage in pH 0.5 was higher than 8 mg each day during the whole reaction. However, an obvious decrease in the Cr(VI) dosage could be observed when the initial pH was higher than 1.0, which was consistent with the low Fe(II) concentration and high Fe(III) precipitation ratio. Sustainable Cr(VI) reduction was only achieved in the pH 0.5 system without exogenetic proton compensation. In addition, it should be noted that the daily reduction dosage increased when inoculated with *A. ferrooxidans*, and the plateau stage was postponed. Figure 1 shows that the Fe(II) concentration in the biological system was lower than that in the chemical system. Based on this fact, it can be speculated that *A. ferrooxidans* activated the reactive sites on pyrite and led to high reduction efficiency. The pyrite-based Cr(VI) reduction efficiency was enhanced by *A. ferrooxidans*, and this promotion effect was most obvious in the pH 1.0–1.5 interval, which might relate to the optimal physiological activity of *A. ferrooxidans* under this pH.

**Figure 5:**
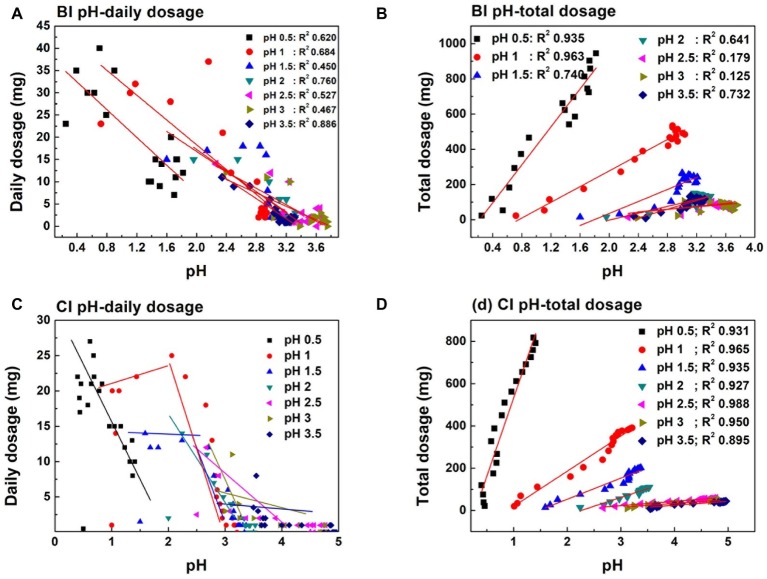
Linear dependence between daily dosage **(A,C)**/total dosage **(B,D)** and pH variation in pH initial adjustment system.

Figures 4E,F exhibits the Cr(VI) reduction capacity in the pH-independent and -stable systems. The reduction capacity of the BI and CI systems decreased with increasing pH. The reduction capacity of the biological system was significantly higher than that of the chemical system. This also verified that reactive site regeneration under *A. ferrooxidans* activity resulted in the promotion of Cr(VI) reduction. The reduction capacity nearly remained unchanged when pH was higher than 2.0 in the proton uncompensated circumstance. The highest Cr(VI) reduction levels reached were 932 mg in the chemical system and 1,113 mg in the biological system under a stable pH in the range 1.0–1.5 (Figures 4E,F). The reduction efficiency of the pH-stable biological system was improved by 4.2, 19.4, 14.7, 72.3, 68.1, and 94.3% compared with the pH-stable chemical system with pH increase. The reduction capacity was not fully consistent with free Fe(II) content in solution, which further confirmed the speculation that reactive sites on pyrite acted as the reductant for Cr(VI). Figure 5 shows the linear dependence between pH variation and daily dosage (A, C)/total dosage (B, D). As aforementioned, Cr(VI) reduction was dependent on multiple factors, which led to a low relevancy between pH and daily dosage. However, a typical linear dependence was exhibited between total dosage and pH. The linear dependency in the CI system was higher than 0.89, and it decreased prominently in the biological system. The complexity of the reaction increased due to interaction between bacteria and pyrite, which resulted in the decreasing relevancy.

### The Relationship Between Reduction Performance and Cr(VI) Dosage

**Figure 6:**
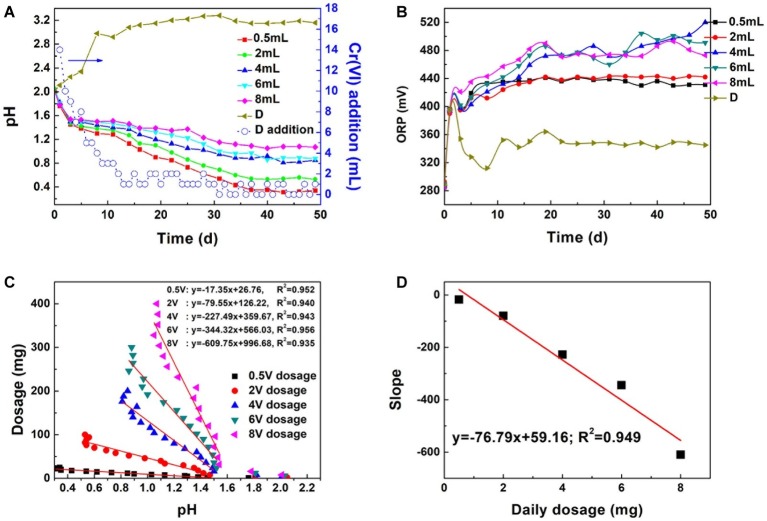
The influence of dosage on pH **(A)** and ORP **(B)**, linear dependence between dosage and system pH **(C)**, linear dependence between slope and daily dosage **(D)**: D means the pH and ORP variation in the maximal Cr(VI) dosing system; **(D)** addition represents Cr(VI) dosing every day.

Besides proton compensation, Cr(VI) dosage was further investigated. Figures 6A,B shows the pH and redox potential change under Cr(VI) dosage gradients. A steady decrease trend can be observed with Cr(VI) addition (0.5–8 mg dosage). The decreasing pH indicated the dissolution of pyrite. Proton produced by bacteria exceeded those consumed in the reduction reaction. Basically, pH was maintained at 0.4, 0.6, 1.0, 1.1, and 1.2 at the plateau (about 37 days) with increasing dosage. In contrast, the pH of the maximal dosing system (D) increased drastically and reached 3.0 on the 10^th^ day. *A. ferrooxidans* cannot be adsorbed onto the pyrite surface tightly in the initial reaction stage (Supplementary Figure S2), so biofilm formation was impossible because of the weak interaction between bacteria and pyrite (Mielke et al., 2003; Zhu et al., 2015). If reductive sulfide and Fe(II) was completely consumed by Cr(VI), it would lead to energy source scarcity and bacteria proliferation stagnation. The amount of Cr(VI) dosing in the maximal dosing system (D) was no higher than 4 mg each day after 10 days, which implied that the biological dissolution effect and active site regeneration activity of *A. ferrooxidans* on pyrite was inhibited. Redox potential presented a different variation trend than did pH. Redox potential reached 480 mV in the 4, 6, and 8 mg dosage systems, and that in the 0.5 and 2 mg systems was lower than in the former, being maintained at approximately 440 mV. In practical applications, Cr(VI) dosage is one of the foremost parameters that should be taken into consideration.

The correlation coefficient between Cr(VI) total dosage and pH reached 0.952, 0.940, 0.943, 0.956, and 0.938, respectively, with increasing dosage (Figures 6C,D). The steady decrease in pH implied that the proton regeneration effect was stronger than the acid consumed in Cr(VI) reduction. The absolute values of the pH-dosage slope were 17.35, 79.55, 227.49, 344.32, and 609.75. The slope increased with dosage. The significant positive correlation between proton consumption ratio and daily dosage illustrated that proton consumption increased with daily dosage. A linear model can be applied in pH prediction to avoid excessive Cr(VI) addition and reduce toxicity to bacteria.

### Pyrite Dissolution and Bacteria Proliferation Under Different Dosages

**Figure 7:**
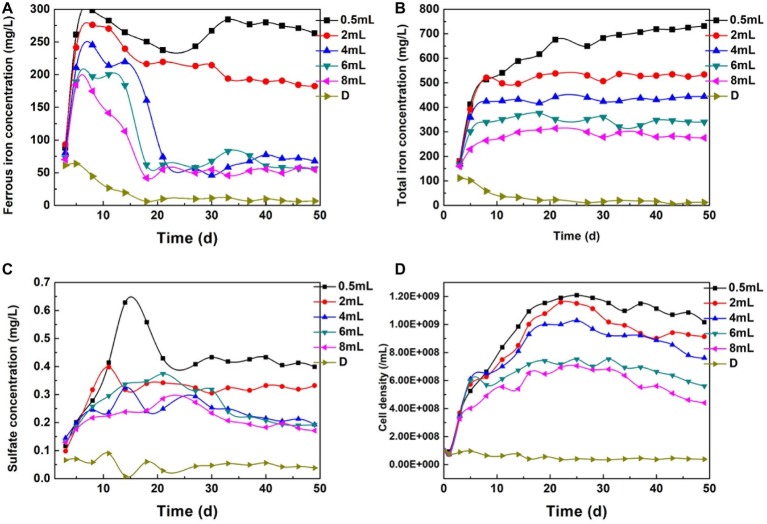
The Change of Fe(II) **(A)**, TFe **(B)**, SO_4_^2−^ **(C)**, and bacterial density **(D)** under different dosage.

Fe(II) and TFe are direct indicators of the complex process that includes biologically and chemically induced pyrite dissolution, Fe(II) oxidation, and iron coprecipitation. Figure 7 shows that Fe(II) decreased continually, and only 26.6 mg/L Fe(II) remained on the 11th day in the D system. In contrast, the Fe(II) concentration in certain dosage systems increased rapidly in the first 10 days, which implied that Fe(II) regeneration exceeded that consumed in Cr(VI) reduction and bacteria metabolism. The SO_4_^2−^ variation trend was similar to the Fe(II) change (Figure 7C). The steadily increasing total iron indicated that the formation of secondary iron minerals and the surface passivation effect were not significant. High Fe(III) iron concentration promoted pyrite dissolution and Fe(II) regeneration (Eq. 4). *A. ferrooxidans* proliferated quickly in the first 20 days (Figure 7D), which indicated that the energy source supply was sufficient. In contrast, bacterial proliferation in the maximal dosing system was basically stagnant due to the high-pH environment and lack of available substrate. These phenomena illustrate that the reduction reaction was sustainable when Cr(VI) reduction and bacteria activity were in a balanced state.

In the maximal dosing system, no bacterial corrosion pits could be found. EDS analysis showed that the Cr, Mg, Si, and P contents reached 3.78, 5.48, 1.24, and 1.79%, respectively (Supplementary Table S1). The surface element composition was consistent with the characteristics of a passivation layer (Stott et al., 2000; He and Traina, 2005). This layer led to the inhibition of the reduction reaction. However, corrosion pits spread through the mineral surface in the fixed dosing systems, and intimate interaction occurred between bacteria and pyrite.

### Morphology and Phase Transformation in pH-Controlled and pH-Non-controlled Systems

**Figure 8:**
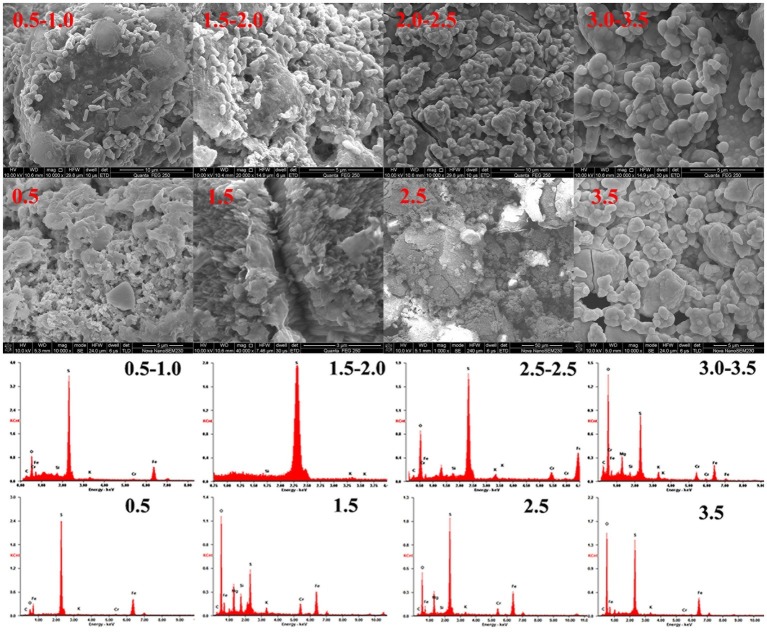
Morphology and EDS spectra of pyrite in an *A. ferrooxidans*-present system.

Figure 8 shows that direct interaction occurred between *A. ferrooxidans* and the mineral. Plentiful cells were attached to the pyrite surface in the BS pH 0.5–1.0 and 1.5–2.0 systems, especially the latter. Corrosion pits spread on the pyrite surface, and extracellular polymeric substances were secreted outside cells. Research has shown that the optimal pH for *A. ferrooxidans* is in the 1.5–2.5 range and that the proliferation and biological activity would be inhibited in a high-pH environment (Meruane and Vargas, 2003). Reduction efficiency increased with decreasing pH in an environment lacking *A. ferrooxidans*, while the highest reduction efficiency of the biological system was achieved in a specific pH interval. *A. ferrooxidans* in the optimal physiological condition accelerated pyrite dissolution and enhanced the corresponding Cr(VI) reduction. A balance between Cr(VI) reduction and biological activity was critical for sustainable reduction. The cell adsorption efficiency decreased markedly when the pH was higher than 2.0, and a passivation film was also formed under this circumstance. This result was also verified by the EDS results (Supplementary Tables S2, S3). The Cr content in BS pH 0.5–1.0 and 1.5–2.0 was 1.33 and 1.09%, respectively, while it increased to 5.32 and 4.03% under pH 2.0–2.5 and 3.0–3.5 conditions. The K content also reached 1.95 and 1.92%. The high K and Cr contents confirmed that a passivation layer formed on the pyrite (Stott et al., 2000; He and Traina, 2005; Cordoba et al., 2009). Corrosion pits also existed on the pyrite surface in the BI pH 0.5 and 1.0 systems, but cell–pyrite interaction was not significant. The Cr content in these four systems was 1.38, 4.46, 5.8, and 7.18%, respectively, with increasing initial pH, much higher than that in the stable system. The interaction between cells and minerals, as well as the passivation film formation, can be regulated through proton compensation.

**Figure 9:**
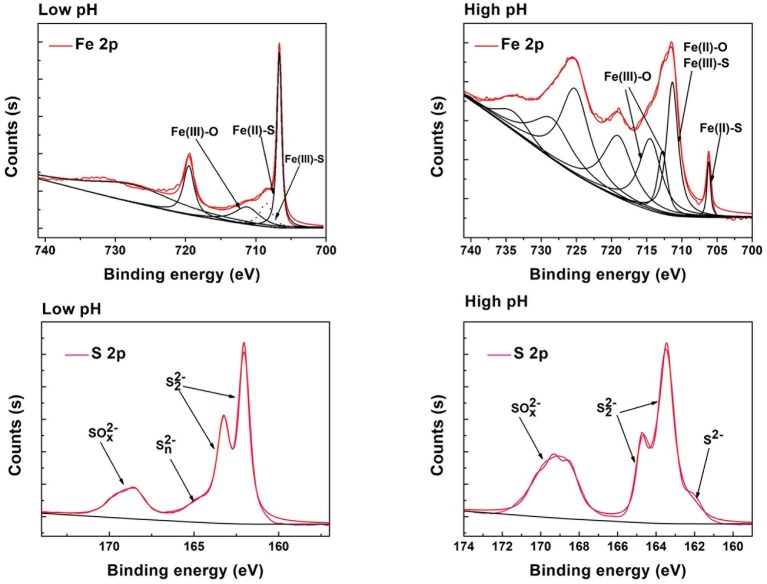
Fitted XPS core level spectra of the S, Fe from different pH environments.

**Figure 10:**
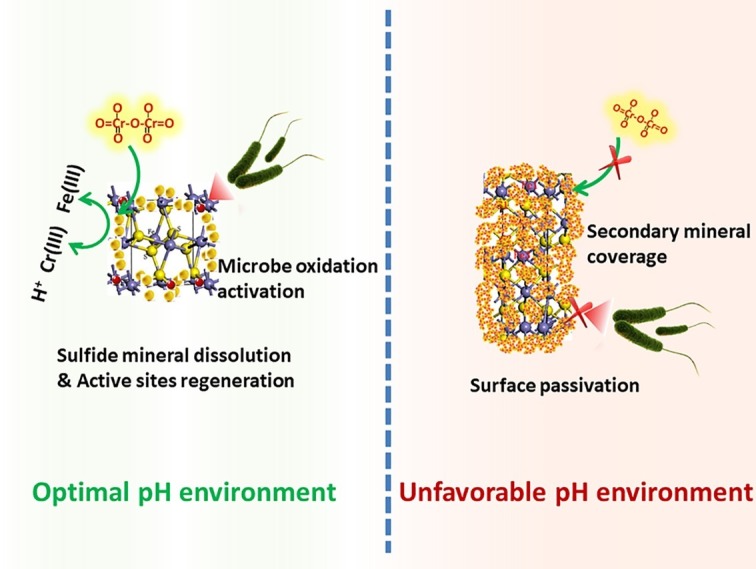
Schematic of Cr(VI) reduction in different pH environments.

XRD analysis (Supplementary Figure S3) was used to identify phase transformation in the different reduction systems. The results showed that the main substance was still pyrite, with trace elemental sulfur, under low-pH conditions (BI/CI pH 0.5, BS/CS 0.5–1.0). As the EDS results showed, there was 39.0% sulfur content in the BI system and 37.07% in the BS system, which also confirmed sulfur formation in the reduction process. Fe(II) oxidized and dissolved from the pyrite lattice under the combined effect of Fe(III)/Cr(VI) and biological oxidization (Mullet et al., 2007). However, S_2_^2−^ was oxidized to elemental sulfur through the thiosulfate pathway. Moreover, sulfur species are inert to abiotic oxidation in acidic environments, so oxidation is exclusively carried out by microorganisms (Schippers et al., 1996; Olson et al., 2003). Elemental sulfur would be accumulated on the bulk mineral if sulfur-oxidizing microorganisms were absent or their activity inhibited. Therefore, *A. ferrooxidans-*dominant sulfur oxidation determined the transformation of reductive sulfide. In contrast, jarosite, hydroxyl iron sulfate, and chromium-iron compounds were identified from the residue in higher pH environments (initial pH system 3.5 and stable pH system 3.0–3.5). Secondary iron minerals resulted in a passivation effect in higher pH environments, which was more significant than the coverage with elemental sulfur in low-pH environments. The above results were also verified by the XPS results (Figure 9). Fe species including Fe(II)-S, Fe(III)-O, and Fe(II)-O/Fe(III)-S existed in both pH environments (Gan et al., 2015; Gong et al., 2017), but there are marked differences between samples. The Fe(II)-S species was the main component of the optimal pH environment pyrite. This confirmed that reactive sites efficiently regenerated, and the pyrite surface was kept “fresh” under this circumstance. However, the Fe(II)-S species was extremely scarce in the high-pH systems and was oxidized to high-valence Fe(III). The main Fe species were Fe(III)-O and Fe(III)-S, which were critical components of the secondary iron minerals. Sulfur intermediate species transformations in different environments were also analyzed. Reduction state S_2_^2−^ and S^2−^, intermediate S_n_^2−^ and S^0^, and SO_x_^2−^ (SO_3_^2−^/SO_4_^2−^) existed on the pyrite. It can be noted that the main difference was the concentration of SO_x_^2−^. The SO_3_^2−^/SO_4_^2−^ species content at the pyrite surface was obviously higher in high-pH environments than that in low-pH systems. The XPS results were in good agreement with those of the other tests. Both the secondary iron minerals and the elemental sulfur formation would inhibit pyrite dissolution, yet the activity of acidophilic bacteria efficiently promoted pyrite dissolution, resulting in proton and reactive site regeneration in Cr(VI) reduction. These biological activities depended on an appropriate physiological pH. The synergistic effect between pyrite and *A. ferrooxidans* can only be achieved in the optimal pH range. Figure 10 summarizes the Cr(VI) reduction mechanisms in different pH environments.

## Conclusions

In this study, the influence of pH and dosage on pyrite-based Cr(VI) reduction efficiency was systematically investigated. The results showed that pyrite based Cr(VI) reduction was a highly proton-dependent process. The Cr(VI) reduction capacity of both BI and CI systems decreased with increasing pH. However, the activity of *A. ferrooxidans* significantly enhanced the Cr(VI) removal efficiency. With increasing pH, the reduction efficiency of pH-stable biological systems was improved by 4.2, 19.4, 14.7, 72.3, 68.1, and 94.3% compared with under pH-stable chemical treatment. The highest reduction efficiency of the biological system was achieved in a specific pH range. Pyrite dissolution and reactive site regeneration were promoted by *A. ferrooxidans*, which resulted in the enhancement of Cr(VI) reduction. The complexity of the pyrite-based reduction system increased due to interaction between bacteria and pyrite, resulting in the decrease in the linear relevancy between pH and Cr(VI) dosage. The passivation effect became more significant with increasing pH, and secondary iron mineral formation will have inhibited the pyrite dissolution. However, the inhibition effect was relieved under the activity of *A. ferrooxidans* due to S/Fe oxidization. A balance between Cr(VI) reduction and biological activity was critical for a sustainable reduction reaction. This research was an extension of the biohydrometallurgy technique in the environmental remediation area.

## Data Availability Statement

The raw data supporting the conclusions of this manuscript will be made available by the authors, without undue reservation, to any qualified researcher.

## Author Contributions

MG conceptualized the study and wrote the manuscript. XL and HW carried the experiment. GQ helped in revising the manuscript.

### Conflict of Interest

The authors declare that the research was conducted in the absence of any commercial or financial relationships that could be construed as a potential conflict of interest.
