# Supplementary material for: Pyrite-Based Cr(VI) Reduction Driven by Chemoautotrophic Acidophilic Bacteria
Source: Front Microbiol. 2020 Feb 7;10:3082. doi: 10.3389/fmicb.2019.03082 (PMC7020336; doi:10.3389/fmicb.2019.03082)
Supplement: Supplementary file 1 [file Data_Sheet_1.docx]

**Pyrite-based Cr(VI) reduction** **driven by chemoautotrophic acidophilic bacteria**

Xinxing Liu^1^, Haiyan Wu^1^, Min Gan^*^, Guanzhou Qiu

*School of Minerals Processing and Bioengineering, Key Laboratory of Biohydrometallurgy of Ministry of Education, Central South University, Changsha 410083, China;*

^1^Xinxing Liu and Haiyan Wu have contributed equally to this work.

*Corresponding authors:

Min Gan, E-mail: ganmin0803@sina.com

School of Minerals Processing and Bioengineering, Key Laboratory of Biohydrometallurgy of Ministry of Education, Central South University, Changsha 410083, China


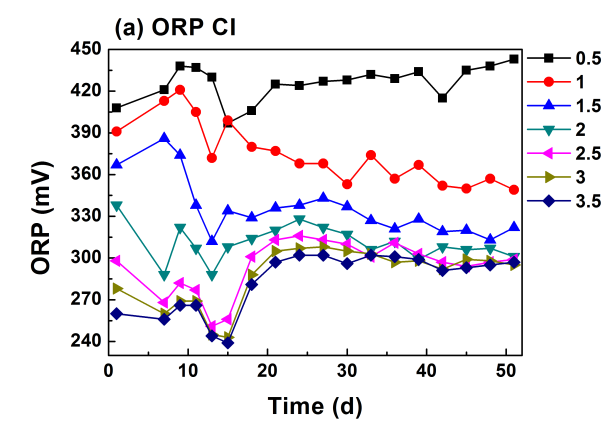

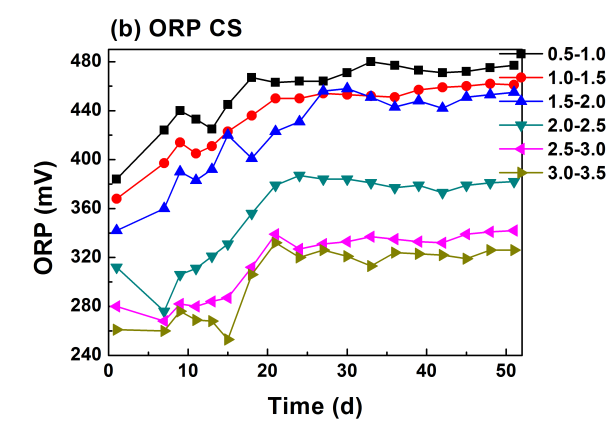

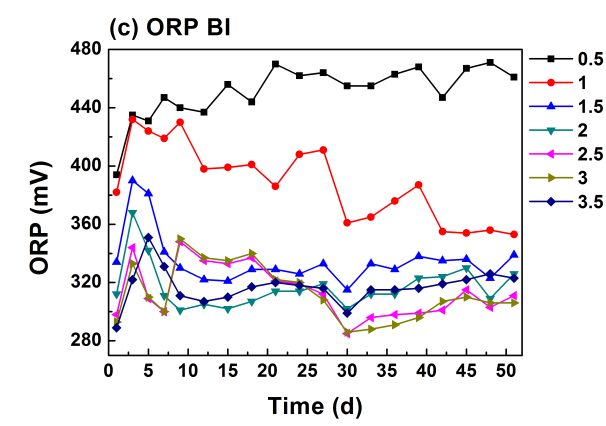

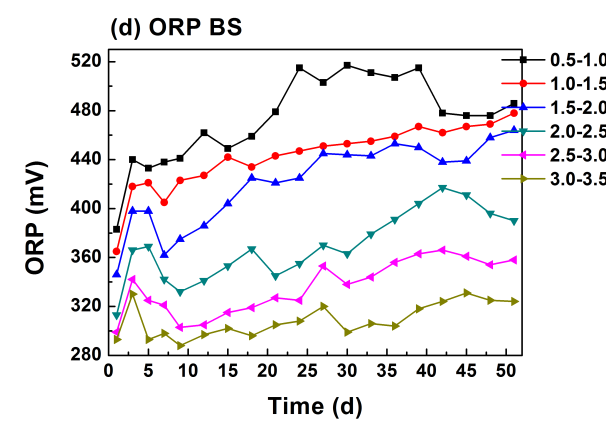


Fig. S1 ORP change in pH stable and independent system


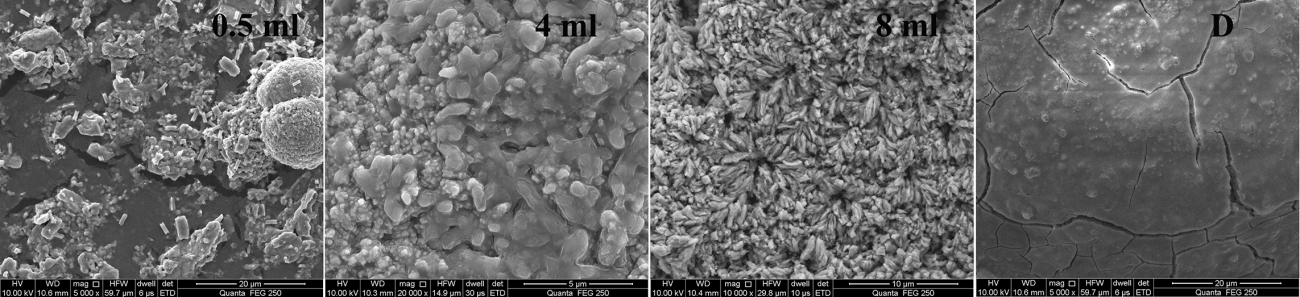


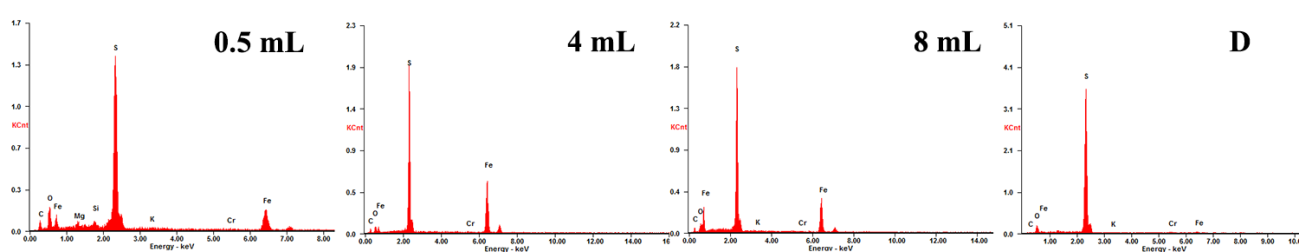


Fig. S2 Morphology and EDS spectra of pyrite in dosage 0.5 mL, 4 mL, 8 mL and maximal dosage











Fig. S3 XRD patterns of the residue from different systems

Table S1 Surface elemental composition of pyrite with dosage in 0.5 mL, 4 mL, 8 mL and maximal dosage

| Dosage | C | O | Mg | Si | P | S | K | Cr | Fe |
| --- | --- | --- | --- | --- | --- | --- | --- | --- | --- |
| 0.5 ml | 5.74 | 64.6 | / | / | / | 17.6 | 0.69 | 0.37 | 11 |
| 4 ml | 10.67 | 14.15 | / | / | / | 54.72 | / | 0.5 | 19.96 |
| 8 ml | 10.09 | 17.3 | / | / | / | 48.72 | 0.49 | 0.96 | 22.44 |
| D | 5.09 | 48.71 | 5.48 | 1.24 | 1.79 | 17.87 | 0.64 | 3.78 | 15.4 |

Table S2 Surface elemental composition of pyrite in *A. ferrooxidans* existing pH stable system

| pH | C | O | Si | S | K | Cr | Fe |
| --- | --- | --- | --- | --- | --- | --- | --- |
| 0.5-1.0 | 14.07 | 22.11 | 0.93 | 37.07 | 1.45 | 1.33 | 23.04 |
| 1.5-2.0 | 12.47 | 15.86 | 0.1 | 37.06 | 0.85 | 1.09 | 32.58 |
| 2.0-2.5 | 11.53 | 27.36 | 0.85 | 24.88 | 1.95 | 5.32 | 28.11 |
| 3.0-3.5 | 22.25 | 44.02 | 0.66 | 12.15 | 1.92 | 4.03 | 11.57 |

Table S3 Surface elemental composition of pyrite in *A. ferrooxidans* existing pH independent system

| pH | C | O | Mg | S |  | K | Cr | Fe |
| --- | --- | --- | --- | --- | --- | --- | --- | --- |
| 0.5 | 13.77 | 38.1 | 1.83 | 39.03 |  | 1.45 | 1.38 | 4.44 |
| 1.5 | 18.77 | 53.43 | 5.74 | 5.78 |  | 0.68 | 4.46 | 11.14 |
| 2.5 | 19.37 | 44.45 | 5.13 | 9.46 |  | 2.07 | 5.8 | 13.28 |
| 3.5 | 25.73 | 28.25 | 3.19 | 25.5 |  | 1.81 | 7.18 | 8.1 |
